# Music intervention improves the physical and mental status for patients with breast cancer: A protocol of randomized controlled trial

**DOI:** 10.1097/MD.0000000000023461

**Published:** 2020-12-04

**Authors:** Xiuting Li, Guangpeng Du, Wei Liu, Fangfei Wang

**Affiliations:** a Department of Radiology; b Department of General Medicine; c Department of Neurology; d Department of Urology, Jinan Central Hospital, Shandong, China.

**Keywords:** breast cancer, mental, music intervention, physical, protocol

## Abstract

**Background:**

Breast cancer is the most familiar cancer and the major cause of the cancer death in women worldwide. The breast cancer patients may suffer from severe mental and physical trauma. At present, there are few studies on the music therapy for patients with breast cancer. The objective of our paper is to assess the effect of music intervention on mental and physical state of breast cancer patients.

**Methods:**

The experiment will be implemented from June 2021 to June 2022 at Jinan Central Hospital. The experiment was granted through the Research Ethics Committee of Jinan Central Hospital (no.08847765). The inclusion criteria requires that the age of female patients ranges from 25 to 65 years old, and the pathological diagnosis of breast cancer requires radical mastectomy (containing extensive radical mastectomy and modified radical mastectomy). Patients who do not like to listen to music or have severe debilitating diseases or are allergic to the sound will be excluded. Patients in the intervention group are given music intervention, and in control group, patients do not receive any information about the music therapy in the period of this study. The primary outcome is quality of life, psychological distress. The secondary outcomes are the heart rate, blood pressure, as well as Visual Analog Scale (VAS).

**Results:**

Table 1 will illustrate the postoperative outcomes after music interventions between groups.

**Conclusion:**

Music intervention can improve the mental and physical health of the breast cancer patients.

**Trial registration:**

This study protocol was registered in Research Registry (researchregistry6168).

## 1 Introduction

Breast cancer is one of the major causes of the death among female patients between 35 and 50 years of age.^[1,2]^ Because of its high incidence rate, huge medical expenses and high mortality rate, it has become a major public health burden.^[3]^ The incidence rate of breast cancer is increasing worldwide and rising even faster in countries with lower rates.^[4]^ According to data from the developed countries, it can be seen that 1 in 8 women is at risk of suffering from breast cancer.^[5]^

The treatment of breast cancer generally begins with surgery, followed by a combination of a variety of adjuvant therapies, including hormone therapy, chemotherapy, and radiotherapy.^[6–8]^ Nevertheless, these advanced treatments are inherently aggressive and increases the exposure of patients to the side-effects of treatment. Female patients with breast cancer, particularly the patients who have undergone radical mastectomy, are facing huge pressure in their life. This includes damaged body images due to the loss of breasts, which can result in enduring and experiencing the negative effects of subsequent unhealthy emotions.

The researches of the impacts of music therapy on the cancer patients with a variety of diagnoses have exhibited many benefits.^[9]^ Listening to the relaxing music is effective in reducing the distress associated with treatment, relaxing, decreasing anxiety, increasing comfort, and reducing nausea and vomiting.^[10]^ Other relevant researches have also indicated that the music therapy is an effective treatment in arousing positive memories and emotions, improving life quality and the quality of sleep, emotion, self-awareness, enhancing mental health, and reducing psychological symptoms (involving fatigue, fear, anxiety, diastolic blood pressure, and worry). It is reported that the music therapy can directly reduce the patients pain through specific social emotional, psychological, and physiological mechanisms.^[11]^ At present, there are few studies on the music therapy for patients with breast cancer. The objective of our paper is to assess the influences of the music intervention on mental and physical state of breast cancer patients.

## 2 Methods

The experiment will be implemented from June 2021 to June 2022 at Jinan Central Hospital. The experiment was granted through the Research Ethics Committee of Jinan Central Hospital (no.08847765) and recorded in research registry (researchregistry6168). Before the registration, the patients who are recruited receive written informed consent.

### 2.1 Inclusion and exclusion criteria

Eighty patients with breast cancer are included in the trial. The inclusion criteria requires that the age of female patients ranges from 25 to 65 years old, and the pathological diagnosis of breast cancer requires radical mastectomy (containing extensive radical mastectomy and modified radical mastectomy). Patients who do not like to listen to music or have severe debilitating diseases or are allergic to the sound will be excluded.

### 2.2 The intervention group

In intervention group, patients are introduced to 5 kinds of music and 100 music names stored in music media library on the MP3 players. Music therapy is provided through the trained researchers. The patients are instructed to choose their favorite music, control the volume of music, and then listen to it through utilizing the headphones linked to MP3 player. The whole intervention time contains hospitalization time after conducting the radical mastectomy and 2 periods of chemotherapy. Patients are asked to listen to the music 3 times a day (30 minutes each time) in the morning, noon, and evening, respectively. During the postoperative hospitalization, if the patients miss listening to music, the researchers encourage her to adhere. Once the patients are discharged from the hospital, they are followed up via a researcher over the phone.

### 2.3 The control group

The patients in control group do not provide any music therapy information in the period of study and only offer the routine care, involving chemotherapy nursing care and breast cancer nursing care.

### 2.4 Outcomes

The main outcome, life quality, is detected via the Functional Assessment Cancer Therapy-General (FACT-G), with a total of 27 items, which utilizes the five-point (0–4) agreement scale.^[12]^ Mental distress is detected through utilizing Hospital Anxiety and Depression Scale (HADS), which is a self-reported measure with a total of 14 items, and contains two 7 subscales to evaluate depression and anxiety.^[13]^ The secondary outcomes are the heart rate, blood pressure, as well as Visual Analog Scale (VAS).

### 2.5 Statistical analysis

The analysis of all the data are conducted with the software of IBM SPSS Statistics for Windows, version 20 (IBM Corp., Armonk, NY, USA). Subsequently, all data obtained are expressed with proper characteristics, for instance, median, mean, percentage and standard deviation. Continuous and categorical variables are analyzed using χ^2^-tests and independent *t* tests, respectively. Intention-to-treat analysis is used for the outcome assessments. *P* value less than .05 indicates that there is statistical significance.

## 3 Results

Table 1 will illustrate the postoperative outcomes after music interventions between groups.

**Table 1:** Postoperative outcomes after music interventions between groups.

Outcomes	Interventiongroup (N = 40)	Control group (N = 40)	*P* value
FACT-G			
Physical Well-Being Subscale			
Social Well-Being Subscale			
Emotional Well-Being Subscale			
Functional Well-Being Subscale			
HADS			
HADS Anxiety Subscale			
HADS Depression Subscale			
Visual analog scale			

FACT-G = Functional Assessment Cancer Therapy-General, HADS = Hospital Anxiety and Depression Scale.

## 4 Discussion

Breast cancer is the most familiar cancer and the major cause of the cancer death in women worldwide.^[14,15]^ The breast cancer patients may suffer from severe mental and physical trauma, involving damaged body images, the loss of breasts, insomnia, fatigue, pain, depression, as well as other negative emotions.^[16]^ These traumas may have a negative impact on the long-term life quality in the survivors of breast cancer. Medication and surgery may be effective, but there may be secondary effects and side effects, resulting in some negative psychological impacts.^[17]^

Patients with breast cancer are more likely to have anxiety and depression symptoms. In turn, depression can cause digestive problems, sleep difficulties, fatigue, lowered immune function, prolonged arousal, morbid anxiety, helplessness as well as pessimism.^[18]^ Severe anxiety may induce patients to experience high blood pressure and increased heart rate. Furthermore, women undergoing breast cancer surgery generally experience pain after operation, which can lead to considerable psychological and physical impairment. The art therapy and cognitive-behavioral therapy are the most familiar methods to manage the psychological state of these patients.^[19,20]^ Among those methods, music intervention is highly recommended. Music therapy is carried out through the well-trained music therapists with various experience in the area of music, it is a process of systematic treatment containing evaluation, treatment, and assessment. Although several articles have reported the application of musical therapy in breast cancer patients, the positive effect remains under debate. This research may provide an evidence regarding the topic.

## 5 Conclusion

Music intervention can improve the mental and physical health of the breast cancer patients.

## Author contributions

Fangfei Wang plans the study design. Wei Liu reviews the study protocol. Guangpeng Du will recruit participants and collect data. Xiuting Li writes the manuscript. All of the authors have read, commented on, and contributed to the submitted manuscript.

**Conceptualization:** Guangpeng Du.

**Data curation:** Wei Liu.

**Funding acquisition:** Fangfei Wang.

**Investigation:** Wei Liu.

**Methodology:** Wei Liu.

**Software:** Xiuting Li.

**Writing – original draft:** Xiuting Li.

**Writing – review & editing:** Fangfei Wang.
